# A non-coding variant in the Kozak sequence of *RARS2* strongly decreases protein levels and causes pontocerebellar hypoplasia

**DOI:** 10.1186/s12920-023-01582-z

**Published:** 2023-06-21

**Authors:** Romain Nicolle, Nami Altin, Karine Siquier-Pernet, Sherlina Salignac, Pierre Blanc, Arnold Munnich, Christine Bole-Feysot, Valérie Malan, Barthélémy Caron, Patrick Nitschké, Isabelle Desguerre, Nathalie Boddaert, Marlène Rio, Antonio Rausell, Vincent Cantagrel

**Affiliations:** 1 Developmental Brain Disorders Laboratory, Université Paris Cité, INSERM UMR1163, Imagine Institute, 75015 Paris, France; 2 Clinical Bioinformatics Laboratory, Université Paris Cité, INSERM UMR 1163, Imagine Institute, Paris, 75015 France; 3 Fédération de Génétique et Médecine Génomique, Service de Médecine Génomique des Maladies Rares, AP-HP, Necker Hospital for Sick Children, Paris, 75015 France; 4 Genomics Platform, Université Paris Cité, INSERM UMR 1163, Imagine Institute, Paris, 75015 France; 5 Bioinformatics Core Facility, Université Paris Cité, INSERM UMR 1163, Imagine Institute, 75015 Paris, France; 6 Département de Neurologie Pédiatrique, AP-HP, Necker Hospital for Sick Children, 75015 Paris, France; 7 Département de Radiologie Pédiatrique, AP-HP, Necker Hospital for Sick Children and Université Paris Cité, INSERM UMR 1163 and INSERM U1299, Imagine Institute, Paris, 75015 France

**Keywords:** Pontocerebellar Hypoplasia, *RARS2*, Kozak, Non-coding variant, Bioinformatic predictions

## Abstract

Bi-allelic variants in the mitochondrial *arginyl-transfer RNA synthetase* (*RARS2*) gene have been involved in early-onset encephalopathies classified as pontocerebellar hypoplasia (PCH) type 6 and in epileptic encephalopathy. A variant (NM_020320.3:c.-2A > G) in the promoter and 5’UTR of the *RARS2* gene has been previously identified in a family with PCH. Only a mild impact of this variant on the mRNA level has been detected. As *RARS2* is non-dosage-sensitive, this observation is not conclusive in regard of the pathogenicity of the variant.

We report and describe here a new patient with the same variant in the *RARS2* gene, at the homozygous state. This patient presents with a clinical phenotype consistent with PCH6 although in the absence of lactic acidosis. In agreement with the previous study, we measured *RARS2* mRNA levels in patient’s fibroblasts and detected a partially preserved gene expression compared to control. Importantly, this variant is located in the Kozak sequence that controls translation initiation. Therefore, we investigated the impact on protein translation using a bioinformatic approach and western blotting. We show here that this variant, additionally to its effect on the transcription, also disrupts the consensus Kozak sequence, and has a major impact on RARS2 protein translation. Through the identification of this additional case and the characterization of the molecular consequences, we clarified the involvement of this Kozak variant in PCH and on protein synthesis. This work also points to the current limitation in the pathogenicity prediction of variants located in the translation initiation region.

**Supplementary Information:**

The online version contains supplementary material available at 10.1186/s12920-023-01582-z.

## Introduction

Pontocerebellar hypoplasia (PCH) is a class of rare neurodegenerative disorders with a pre-natal onset and characterized by degeneration or hypoplasia of the pons and the cerebellum. PCH is further classified into 17 subtypes currently based on the underlying neuropathological features and genetic causes and new subtypes are regularly described [1–5]. These subtypes have an autosomal recessive inheritance, with typical restricted development thereby leading to severe intellectual and motor function impairments, epilepsy and frequently death during childhood [6–9]. Among the genetic causes, variants in t-RNA splicing endonuclease (TSEN) complex genes have been prevalently reported [10–14]. *TSEN54*, *TSEN2*, *TSEN34*, and *CLP1* genes encode members or associated factors of this TSEN complex and variants in those genes have been associated with 6 PCH subtypes: PCH2A (OMIM #277470), PCH2B (OMIM #608753), PCH2C (OMIM #608754), PCH4 (OMIM #225753), PCH5 (OMIM #610204) and PCH10 (OMIM #615803). Further, RNA processing defects due to variants in *EXOSC3*, *EXOSC8* and *EXOSC9* contribute to pathogenesis of PCH1 [15–17]. Hence, defects in RNA metabolism or protein translation can be considered as a common mechanistic factor behind most PCH subtypes.

PCH6 is usually considered as a mitochondrial disease due to variants in the gene encoding for mitochondrial arginyl-transfer RNA (tRNA) synthetase (*RARS2*). RARS2 protein is involved in the addition of arginine to tRNA, thus allowing translation of mitochondrial proteins. Splice site, nonsense, or missense variants on *RARS2* gene have been reported in more than 50 cases, with PCH estimated to be present in half of them [18–23].

There are two main overlapping clinical conditions associated with biallelic pathogenic *RARS2* variants: (i) PCH6 that was initially described and (ii) early onset epileptic encephalopathy without typical PCH on MRI [18, 24, 25]. In the former case, the main clinical features include hypotonia, microcephaly, developmental delay, atrophy of cerebellum and cerebrum as well as high lactate blood-levels. They can be present at birth with hypotonia, lethargy and poor sucking but can also appear during the first months of life, and even though epilepsy is often present, it is not always the first symptom [18, 19, 21]. In the latter case, on the contrary, the main clinical features are seizures occurring in the first weeks of life leading to severe neonatal developmental delay and epileptic encephalopathy [23]. It is worth noting that in some cases, the neurodevelopment can be normal for several months allowing the patients to learn to walk and even to say some words, before a slowing and a regression of cognitive development with loss of milestones [23].

For some of these variants, the consequence on the enzyme activity or *RARS2* mRNA levels have been investigated and an impact at the transcriptional level has been detected multiple times [21, 23]. Most of pathogenic variants that have been reported are missense and some of them are recurrent [23, 26]. Despite the numerous reported variants, it hasn’t been possible to establish precise genotype–phenotype correlation [19, 23, 27].

The identification of disease-causing non-coding variants is a challenging task based on in silico and functional genome annotations. Li et al. reported a variant in the promoter and 5’ untranslated region (UTR) of *RARS2* gene (NM_020320.3: c.-2A > G) as a potential cause of PCH in a single family with two affected siblings [28]. The authors investigated the impact of this variant on the promoter activity and identified a ~ 40% decrease in mRNA levels. *RARS2* has a null pLI score (i.e. the probability of being Loss of function Intolerant; gnomAD v.2.1.1) but a LOEUF score of 0.92, indicating that this gene is slightly intolerant to loss-of-function variants and is not among the dosage sensitive genes [29]. This observation is not unusual for recessive genes, and is consistent with the healthy status of the parents of the *RARS2* patients who are carriers of a severe loss-of-function allele (i.e., likely null) [19, 30]. Consequently, a decrease of about 50%, or less, in the quantity of the *RARS2* transcript’s level is not likely to be sufficient to cause a clinical condition and further characterization of the identified variant is needed to fully demonstrate causality.

This variant has also been reported in an infant with a compatible neurological phenotype although as a compound heterozygous and with limited clinical information [31]. In the present study, we identify a new PCH case with a homozygous variant in the 5’UTR of the *RARS2* gene. We demonstrate that this variant, previously described by Li et al., does not only impact gene transcription but also has an important and direct effect on protein production, hence confirming its pathogenicity. We also further compare the clinical severity, and discuss this finding in light of the bioinformatics predictions and previously described, clinically relevant, variants located in the Kozak sequence.

## Materials and methods

### Patient recruitment and sequencing

The family included in this study was referred to the departments of pediatric neurology and genetics of the Necker Enfants Malades Hospital. High-resolution karyotype, array-comparative genomic hybridization (aCGH) (Agilent 60 K) was performed. Informed consents have been obtained both from the participants and the legal representatives of the children. Sequencing was performed with a custom gene panel including 72 genes involved in early-onset cerebellar atrophy or PCH as previously described [32]. Briefly, genomic DNA libraries were generated using SureSelectXT Library PrepKit (Agilent) and the Ovation Ultralow System V2 (NuGen) according to the suppliers’ recommendations. All exons and 25 base pairs intronic flanking sequences of the 72 selected genes were captured by hybridization with biotinylated complementary 120-bp RNA baits designed with SureSelect SureDesign software. Paired-end sequences were mapped on the human reference genome (NCBI build37/ hg19 version) using the Burrows-Wheeler Aligner. Downstream processing was carried out with the Genome Analysis Toolkit (GATK), SAMtools, and Picard. Sanger sequencing was performed on the patient and parents’ blood samples using the 3500xL Genetic Analyzer (Applied Biosystems).

### Fibroblast culture, RNA extraction and quantitative RT-PCR

All human cell culture and storage protocols were performed with approval from French Research Ministry (DC 2015–2595) and the family provided written consent. Human primary fibroblasts were cultured in Dulbecco’s modified Eagle medium (DMEM) (11965092, Gibco) supplemented with 10% Fetal Bovine Serum (FBS) (16000044, Gibco) and 1% Penicillin–Streptomycin in a humidified incubator (5% ${CO}_{2}$, 37 °C). Total RNA extraction was performed from cell culture pellet using TriZol (ThermoFischer, 15596026) according to the supplier's instructions. The purification of the RNA was done with the RNeasy Mini Kit (Qiagen, 74104). Reverse transcription of the total RNAs was performed using SuperScript II reverse transcriptase (ThermoFisher, 18064022) according to the manufacturer's recommendations. Quantitative PCR was performed with the SYBR Green PCR Master Mix reagent (ThermoFisher, 4364346) on an Applied Biosystems One Step Plus real-time PCR system (Applied Biosystems, 4376600). *GAPDH* was chosen as a reference gene to normalize the results between different tissues and cell line. The relative mRNA levels of *RARS2* were determined by the ΔΔCt method.

### Western blotting

Protein extraction, quantification, separation in gel electrophoresis and transfer were performed using standard procedure [3]. Nitrocellulose membranes were blocked in Odyssey-TM Blocking Buffer (927–50003, LICOR) for 1 h and further incubated overnight at 4 °C with the following primary antibodies diluted in OdysseyTM Blocking Buffer: anti-RARS2 (1:16000, ab230274, Abcam), Anti-β Actin (1:10000, AM4302, Invitrogen). After three washes with 0.2% PBST, the membranes were then incubated for 1 h at room temperature with IRDye-coupled (1:10000, 925–68070, 926–32211, LICOR) secondary antibodies. After three washes with 0.2% PBST, the membranes were processed with Odyssey CLx imaging system (LICOR) and the quantification analysis was performed with Image Studio Lite Ver 5.2, with the default parameters and using β Actin for normalization. Detection and quantification of RARS2 protein level in patient and control was repeated in two independent experiments with comparable results (Supplementary Fig. 1D-F).

### Bioinformatic analysis

We used three complementary bioinformatic predictive tools to assess the pathogenicity of the non-coding genetic variants: NCBoost [33], DeepSEA [34] and TITER [35].

NCBoost is a pathogenicity score for non-coding variants based on supervised learning on manually curated sets of pathogenic and non-pathogenic variants in non-coding regions [33]. NCBoost exploits a diverse range of features including: interspecies sequence conservation, recent and ongoing natural selection signals in humans and epigenetic features. The output of this score gives the likeliness that the genomic position is intolerant to variation without precising its impact on transcription or translation.

DeepSEA is a deep learning-based algorithm framework that predicts the chromatin effects of sequence alterations with single-nucleotide sensitivity. The model has been trained to predict chromatin features like transcription factors binding, DNAse I hypersensitive sites and histone marks [34]. DeepSEA was used in the context of this work to evaluate the transcriptional consequences of genetic variants.

TITER is a deep learning-based framework designed to predict translation initiation sites and translation efficiency by taking into accounts the putative START codon and features of the 200 nucleotides surrounding it [35]. TITER has been trained using global translation initiation sequencing (GTI-seq) and quantitative translation initiation sequencing (QTI-seq) data. Its main purpose is to identify the effective Translation Initiation Site (TIS) by scoring several putative START codons, both in the 5’UTR and in the coding sequence, based on the annotated START codon.

## Results

### Case report

The proband was the first child of distantly related Turkish parents and was delivered at full term following a pregnancy monitored for an increased nuchal translucency. At birth, physical examination was normal: weight was 3,320 g (28th percentile(p)), length 50 cm (28th p) and head circumference 33.5 cm (17th p) (see Table 1).

**Table 1:** Clinical comparison between our Patient and the two siblings previously reported by Li et al. [28]

	**CASE A**	**CASE B**	**CASE C**
Publication	Ours	Li et al., 2015 (Pt 10) [28]	Li et al., 2015 (Pt 11)[28]
Patient (sex, age at study)	A (M, 17 y, died)	Pt 10 (M, 4,5 years)	Pt 11 (F, 18 months)
Epilepsy	generalized tonic clonic seizure	partial epilepsy	NA
Seizures onset	16 months	9 months	NA
First seizure type	generalized tonic clonic seizure	not specified	NA
Subsequent seizures	focal to bilateral tonic clonic seizure, status epilepticus	refractory partial epilepsy	NA
Pharmacoresistant	Yes	Yes	NA
Developmental concern (age of the first symptoms)	4–5 months	9 months	6 months (hypotonia)
Regression (age)	No, never learnt to walk nor to stand without assistance	Yes (9 months): loss of milestones	No, but did not gain milestones in infancy
Neurological examination	Spastic hypertonia of the 4 limbs, prominent in lower limbs and axial hypotonia. Permanently open mouth	4.5 years: axial hypotonia, distal hypertonia, cannot sit nor stand, amblyopia	at 18 months: hypotonia, can sit with support, abnormal abduction of the eyes
Microcephaly	Yes	No	Yes
Movement disorder	Orofacial dyskinesias, myoclonus, dystonia	dysconjugate eye movements	abnormal abduction of the eyes
MRI	pontocerebellar hypoplasia, dragonfly-like cerebellar pattern, no lactate peak (at 6 years)	PCH, prominent subarachnoid space overlying frontotemporal convexities	cerebellar hypoplasia
Metabolic abnormalities	lactate: 2.40 mmol/l (N < 1.8 mmol/l) pyruvate: 0.13 mmol/l (N: < 0.17 mmol/l)	NA	NA

In early infancy (4–5 months), he showed developmental delay (HP:0012758) with generalized hypotonia (HP:0001290). Brain CT scan revealed global cerebellar hypoplasia (prominent on the vermis) with a supratentorial myelination delay (HP:0012448). The brain MRI performed at 2 years of age found cerebellar hypoplasia without atrophy, an arachnoid cyst (HP:0100702) of the posterior fossa, and myelination abnormalities.

He presented with a first epileptic seizure (HP:0001250) at 16 months and a treatment was introduced. Due to recurrent episodes of focal and bilateral tonic clonic seizures (HP:0002266 and HP:0002069) as well as status epilepticus (HP:0002133), a triple-therapy treatment was needed at 4 years and 6 months. At 3 years and 6 months, growth parameters were weight 16 kg (- 2 SD), height 94 cm (- 1 SD) and head circumference 48 cm (- 2 SD). He presented with axial hypotonia (HP:0009062) and peripheral hypertonia (HP:0002509) with extrapyramidal muscular rigidity (HP:0007076) prominent in the lower limbs and dystonia (HP:0001332). He also presented with orofacial dyskinesias (HP:0002310) and glossoptosis (HP:0000162).

Different metabolic diseases were tested (including glycogenosis, neurolipidosis, mucopolysaccaridosis, glycoproteinosis, mucolipidosis, CDG) but the results were negative. The mitochondrial function was normal even though the complex I activity was near the lower range. The lactate blood level was above the normal at 2.40 mmol/l (for a normal value < 1.8 mmol/l) whereas the pyruvate blood level was normal (0.13 mmol/l for a normal value < 0.17 mmol/l). Therefore, the lactate:pyruvate ratio was slightly elevated at 18 (for a normal value between 6 and 14).

**Fig. 1:**
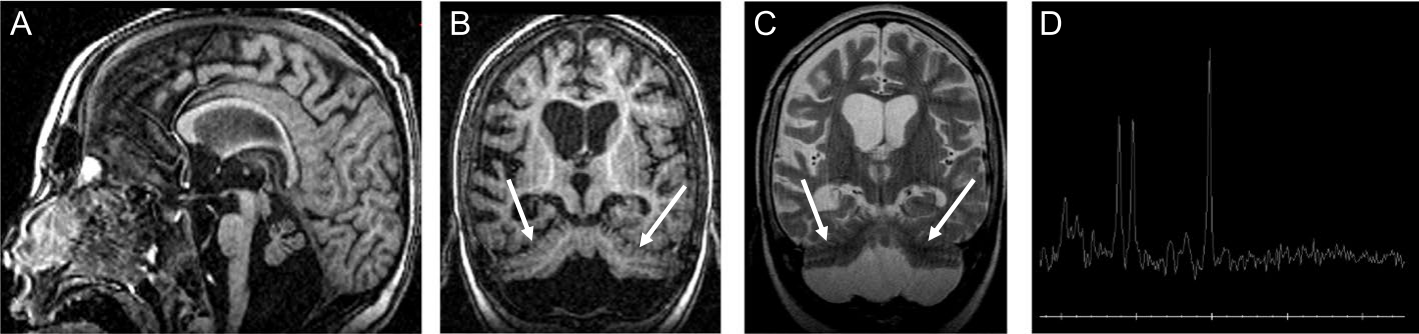
Patient brain MRI at 6 years. **A** Sagittal T1-weighted image showing pontocerebellar hypoplasia. **B** **and** **C** Coronal T1 and T2 weighted images with the "dragonfly-like" cerebellar pattern (flattening and severely reduced size of the cerebellar hemispheres with relative sparring of the vermis) indicated by the white arrows. **D** MR spectroscopy showing no lactate peak

At 6 years, the brain MRI found a pontocerebellar hypoplasia (HP:0001321) with a dragonfly-like cerebellar pattern (Fig. 1B and C, white arrows) associated with attenuated pons (Fig. 1A-C). A supra-tentorial atrophy was also detected without lactate peak (Fig. 1D).

At 7 years, he had microcephaly (head circumference at—2 SD, HP:0000252) and still presented with a severe neurodevelopmental delay without progression nor regression. He still had no voluntary grasping and only few spontaneous movements. He had spastic hypertonia of the four limbs (HP:0002509), prominent in the lower limbs and axial hypotonia with a permanently open mouth (HP:0000194). He never learnt to walk or to stand without assistance and had skeletal muscle atrophy (HP:0003202) and musculotendinous retractions (HP:0031462) caused by limited mobilization. He underwent several orthopedic surgeries including posterior arthrodesis at the age of 12 years for spinal deformities (HP:0008443). Since childhood, he had recurrent respiratory infections (HP:0002205) and deglutition impairments (HP:0002015) leading to feeding difficulties (HP:0011968). He had recurrent pneumonia (HP:0006532) in a context of dysphagia and gastroesophageal reflux (HP:0002020) and a percutaneous endoscopic gastrotomy tube was inserted at the age of 8 years (HP:0011471). He was hospitalized at the age of 17 years for respiratory failure (HP:0002878) in a context of acute infectious pneumonia (HP:0011949) requiring oxygen and antibiotherapy. A decision to limit treatment was made and the patient died a few days later.

### Gene panel sequencing results

**Fig. 2:**
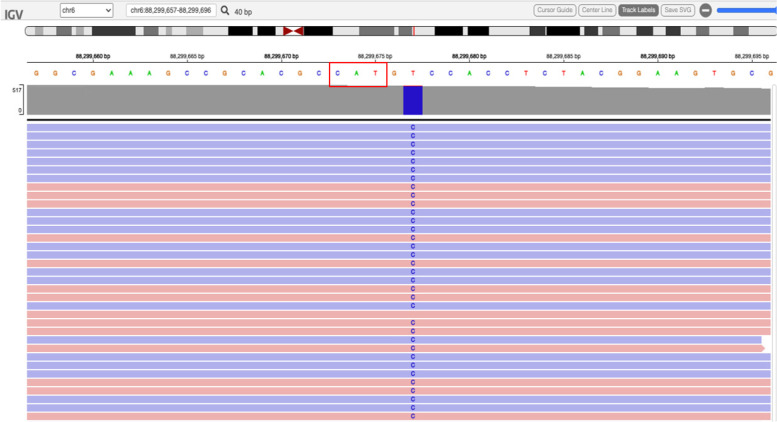
IGV (Integrative Genomics Viewer) track of the variation. The variant NC_000006.11:g.88299677 T > C (NM_020320.3:c.-2A > G) has been detected in a homozygous state in the Patient. The reverse complement sequence is shown

To define an aetiologic diagnosis for the neurological disorder of the affected individual, we used a custom gene panel as a primary test that includes the most common genes involved in early-onset cerebellar atrophy and PCH, as described in Chemin et al. [32]. Using a deep sequencing with a mean targeted coverage of 430X and close to 100% of the targeted DNA covered at 30X, we did not identify rare and potentially damaging coding variants in known PCH genes. Re-analysis of this panel result assessed a homozygous variant (NM_020320.3: c.-2A > G) in the 5'UTR of the *RARS2* gene (Fig. 2). This variant is located two bases upstream of the START codon in the Kozak sequence. The analysis of the affected nucleotide with GERP and PhyloP did not support a strong conservation across the evaluated species with negative conservation scores of -2.32 and -0.87287, respectively. This variant had been previously reported in a homozygous state in two siblings presenting with pontocerebellar hypoplasia [28]. Sanger sequencing confirmed that the parents were both heterozygous carriers of this variant.

### In silico analysis of conservation, transcription and translation efficacy

In order to further evaluate the pathogenicity potential of this variant, we used three complementary bioinformatics tools (Methods). NCBoost evaluates the conservation of sequence across species and within humans [33] and ranked the pathogenicity potential of the altered genomic position among the top 17% (i.e. score of 0.122, rank-percentile of 0.831 for this chromosome) of all cis-proximal non-coding genomic positions for chromosome 6. To estimate the functional impact of this variant at the chromatin level, we used DeepSEA [34] to predict an impact on transcription and TITER [35] to predict consequences on translation.

The DeepSEA functional significance score of the variant is 5.4e-03, which suggests a significant alteration at the transcriptional level, in line with previous observations [28]. More specifically, DeepSEA predictions point at a severe alteration of the binding of the factors YY1 and REST/NRSF that would severely hinder transcription. YY1, a transcription factor with both activator and repressor activities is widely involved in development [36] and REST/NRSF (RE1-silencing transcription factor/Neuron-restrictive silencer factor) is a transcriptional repressor which plays a key role in non-neuronal cells but is also involved in neural cell survival [37].

Then, we used TITER to further investigate a potential additional effect of this variant on the efficacity of the Kozak sequence. The scores for the wild-type sequence and in presence of the NM_020320.3:c.-2A > G variant are nearly identical, 3.4433 and 3.3865 respectively. Thus, this variant is not predicted by TITER to alter translation efficiency.

### RARS2 transcript and protein levels in fibroblasts

**Fig. 3:**
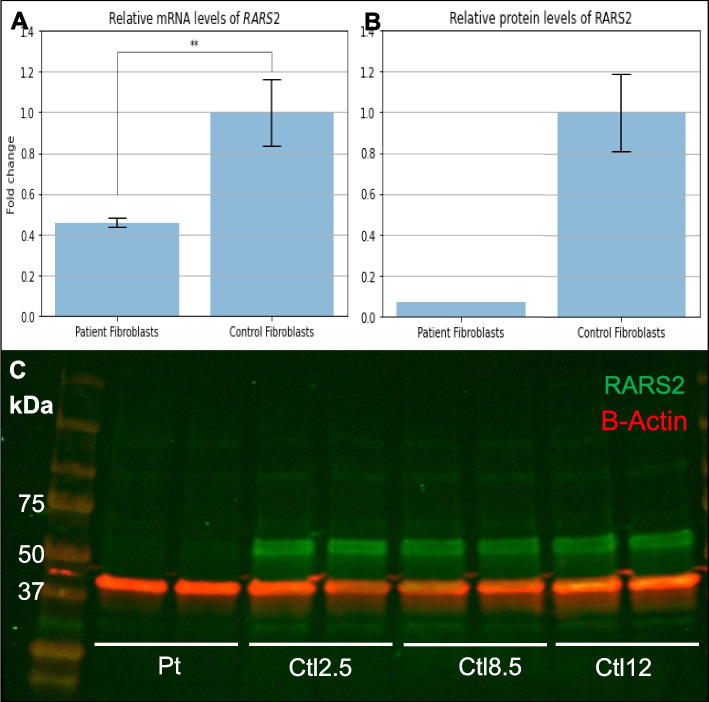
*RARS2* mRNA levels and protein levels on fibroblasts. **A** Real-time PCR performed on the RNA extracted from fibroblasts. The *RARS2* mRNA level in the Patient's fibroblasts is only ~ 46% of the Control's. The statistical analysis was performed using the SciPy (version 1.9.3) package from the Python programming language (version 3.9.15). The *RARS2* mRNA levels were compared to those of *GAPDH* by the ΔΔCt method and based on four cell culture replicates and then three technical replicates for each genotype. The Welch’s test (corrected t-test for unequal variances) was performed on the log10 of the fold change. **B** Quantification of the RARS2 protein levels from the Western blot in 3C and showing a 92.98% decrease in the Patient’s fibroblasts compared to Control’s. **C** Western blotting of RARS2 Patient and Control’s cell lysate using RARS2 (green) antibody. β Actin serves as a loading control. Two technical duplicates were loaded for the Patient’s cell lysate as well as for the three different Controls’. The Western blot displayed here has been cropped and the full-length version is presented in Supplementary Fig. 1. Pt = Patient, Ctl = Control

According to the Ensembl genome browser, there is only one reliable transcript that is well supported by mRNA sequences (with Transcript Support level 1 label), coding for the 578 amino-acids/65 kDa RARS2 protein and no additional Kozak sequence is detected. The impact of the Kozak variant on this transcript was studied by real-time PCR performed on mRNA extracted from fibroblast cultures for both the patient and a control. *RARS2* mRNA levels in the patient cells were significantly decreased by ~ 54% ($p=0.0027$, Fig. 3A). This change is comparable with the ~ 30% and ~ 50% decreases previously detected in peripheral blood from siblings carriers of the same variant [28]. With this partially preserved *RARS2* transcription in mind, we investigated the impact on the protein levels, using Western blot. Strikingly, protein levels were extremely decreased in the patient's fibroblasts, with only a faint residual band quantified and normalized as a ~ 93% decrease compared to control samples (Fig. 3B, C).

## Discussion

We report here a new patient harboring a previously described non-coding variant, NM_020320.3:c.-2A > G, in a homozygous state, in the *RARS2* gene. Although this variant is located in the Kozak sequence, an impact on protein levels was not previously investigated. Using in silico predictions and in vitro analyses, we studied the effects of this single nucleotide variant. Our results identified a combined effect at the transcriptional and translational levels showing that the protein levels are almost abolished in patient’s fibroblasts.

More than 50 patients carrying biallelic variants in the *RARS2* gene have been described with a wide range of clinical manifestations [18–23]. The most common features are developmental delay, seizures, progressive microcephaly, elevated lactate, atrophy/hypoplasia of cerebellar hemispheres and cerebral atrophy [24]. The clinical case described here presented with a phenotype very similar to previously reported patients harboring homozygous deleterious variants[18] or the cases identified by Li et al. with the same variant (NM_020320.3:c.-2A > G) [28], although in the absence of clear lactic acidosis. The overall clinical similarity, with the functional work, are definitive arguments to define this variant as a causative and suggest that this type of severe loss-function allele is associated with a PCH phenotype. An additional patient carrying the same allele was previously described [31]. For this case, compound heterozygous variants in *RARS2* were detected with the Kozak variant (i.e., NM_020320.3:c.-2A > G) inherited from the mother, and a frameshift variant inherited from the father. However, the young age of the patient (i.e., less than a year old) and the possible contribution of other variants to the neurological phenotype are important limitations to interpret the impact of the Kozak variant for this specific case.

Interestingly, a recent report showed that even though *RARS2* is involved in protein translation in the mitochondria, the alteration of the energy metabolism may not be as central to the phenotype as previously reported [27]. The authors showed that at the cellular level, mitochondrial energetic metabolism defects are not consistently detected. Key clinical features include severe neurodevelopmental disorder with early onset-encephalopathy, intractable epilepsy and progressive neurological impairment. They also report some common craniofacial dysmorphism including epicanthus, faint eyebrows, a high nasal bridge, a bulbous and high nose tip, cupid lips, prominent ears and bilateral frontal depression. The Dragonfly pattern on MRI can also be found in PCH1B, 2A, 2B, 2C, 4, 5, and 9 in addition of PCH6 [38]. Even though the seizures are common in this phenotype, the severity is variable and the patients can present with early onset epileptic encephalopathy, even without PCH [22, 23, 39–43]. This gene has also recently been involved in a Finnish cohort of hereditary ataxia [44]. So far, no clear genotype–phenotype correlation could be established. A recent review of the different *RARS2*-related clinical manifestations showed that, even though more than 60% of reported variants are missense, a vast majority of these variations (i.e. ~ 85%) are predicted to or have been proved to alter splicing or gene expression [23]. Consequently, investigation of the gene but also the protein levels in patient’s cells are likely to have a critical importance to decipher the clinical consequences of these variants.

Nucleotides flanking the START codon are necessary to allow its proper identification as the translation initiation site (TIS) by the small subunit of the ribosome. In eukaryotes, the conserved motif allowing the fixation of the small unit of the ribosome is the Kozak consensus sequence: 5'-GCCRCC**AUG**GCG-3', with the initiation codon in bold and the R at the position -3 representing a purine nucleotide [45]. This sequence is highly conserved in eukaryotes, especially across vertebrates. In vitro studies have revealed that the most critical nucleotides are the -3R and the + 4G [46]. Variations from the consensus Kozak sequence can have an impact on the efficiency of the translation. As an example, if one of these critical nucleotides is substituted, the translation decreases five-fold to ten-fold [47]. In our case, the nucleotide mutated is in -2 of the Kozak sequence, which is less documented as being a critical position for translation efficiency. However, this nucleotide (A or C) which is in contact with the translation initiation factor eIF2 is among the most conserved among the Kozak consensus sequences of the vertebrates [46]. Interestingly, using dinucleotide position weight matrix analysis Noderer et al. detected a significant influence of the position -2 that could balance less favorable nucleotides at another position [48].

The prediction software TITER doesn’t predict a difference in RARS2 translation efficacy in the presence of the Kozak variant, contrasting with the severe effect observed in vitro. This difference could be related to the sequence upstream of the Kozak sequence in the *RARS2* gene which might be atypical compared to the dataset that was used to train the neural network underlying TITER’s predictions. Potentially, it can be related to *RARS2* promoter localization that overlaps with the gene START codon and can generate a specially short 5’UTR of 30 nt (NM_020320.3) compared to the median 5’UTR size in human of ~ 150 nt [49]. Interestingly, a translation initiation element distinct from the typical Kozak has been identified in mRNAs with very short 5'UTR and that also regulates transcription [50]. Although not present in *RARS2* as such, this element illustrates the existence of non-typical translation initiation site that can complexify the prediction analysis of these sequences.

Currently, only a few patients harboring variants in the Kozak sequence have been described in the literature and for two of them functional work supports pathogenicity in monogenic context. The first one is an heterozygous Kozak variant located in the -6 nucleotide in *HBB*, associated with another pathogenic variant and responsible for beta-thalassemia in an Italian family [51]. The second one is a substitution also in the -6 nucleotide in the *GATA4* gene that is causing atrial septal defects with an autosomal dominant heredity [52]. Additionally, multiple variants have been described as risk or modifier alleles. A Kozak variant located in the -5 nucleotide of *GP1BA*, coding a component of the GP Ib-IX-V receptor complex has been considered a susceptibility factor for the development of cardiovascular disease [53, 54]. The NM_000505.3:c.-4 T > C transition in the Coagulation factor XII (*F12)* gene is a common polymorphism which is considered to be disease-modifier in hereditary angioedema [55]. The NM_001154.4:c.-1C > T transition in the *ANXA5* gene slightly increases the risk of myocardial infarction in men [56]. A variant at the position -2 in one isoform of the *GRM3* gene (rs148754219) has been considered a risk factor for bipolar disorder [57]. Finally, The SNP rs11545028 (NM_016373.4:c.-5C > T) in the *WWOX* gene is associated with oral cancer risk [58]. In that case, the variant is also located in the gene promoter, and impacts transcription and translation.

Most of the 20 Kozak variants reported in the ClinVar database (see Supplementary Table 1) are interpreted as variants of unknown significance. The likely underrepresentation of the Kozak variants in the medical literature could be related to their annotation as “non-coding variant” when located in the 5’UTR or as missense variants when affecting the nucleotides + 4 to + 6.

Altogether, these observations further stress the need of improved tools to analyze these regions as they are critical for gene regulation and disrupted by disease-causing variants.

## Conclusion

Through the identification of this additional case, this study validates the homozygous *RARS2* variant NM_020320.3:c.-2A- > G as the molecular cause for this severe encephalopathy. Then, we clarified the consequence of this variant on transcription and on protein synthesis. This work suggests that some variants located in the Kozak sequence, including the -2 position, might impact protein translation in a more drastic way than expected based on current in silico predictions.

## Supplementary Information

**Additional file 1:** **Supplementary Figure 1.** Original, full-length version of the Western Blots with RARS2 antibody.

**Additional file 2. **

## Data Availability

The genetic variation analysed during the current study had already been previously reported in the ClinVar repository (https://www.ncbi.nlm.nih.gov/clinvar/variation/802251/).
