# Supplementary material for: A non-coding variant in the Kozak sequence of RARS2 strongly decreases protein levels and causes pontocerebellar hypoplasia
Source: BMC Med Genomics. 2023 Jun 21;16:143. doi: 10.1186/s12920-023-01582-z (PMC10283289; doi:10.1186/s12920-023-01582-z)
Supplement: Supplementary file 1 — Additional file 1: Supplementary Figure 1. Original, full-length version of the Western Blots with RARS2 antibody. [file 12920_2023_1582_MOESM1_ESM.pdf]

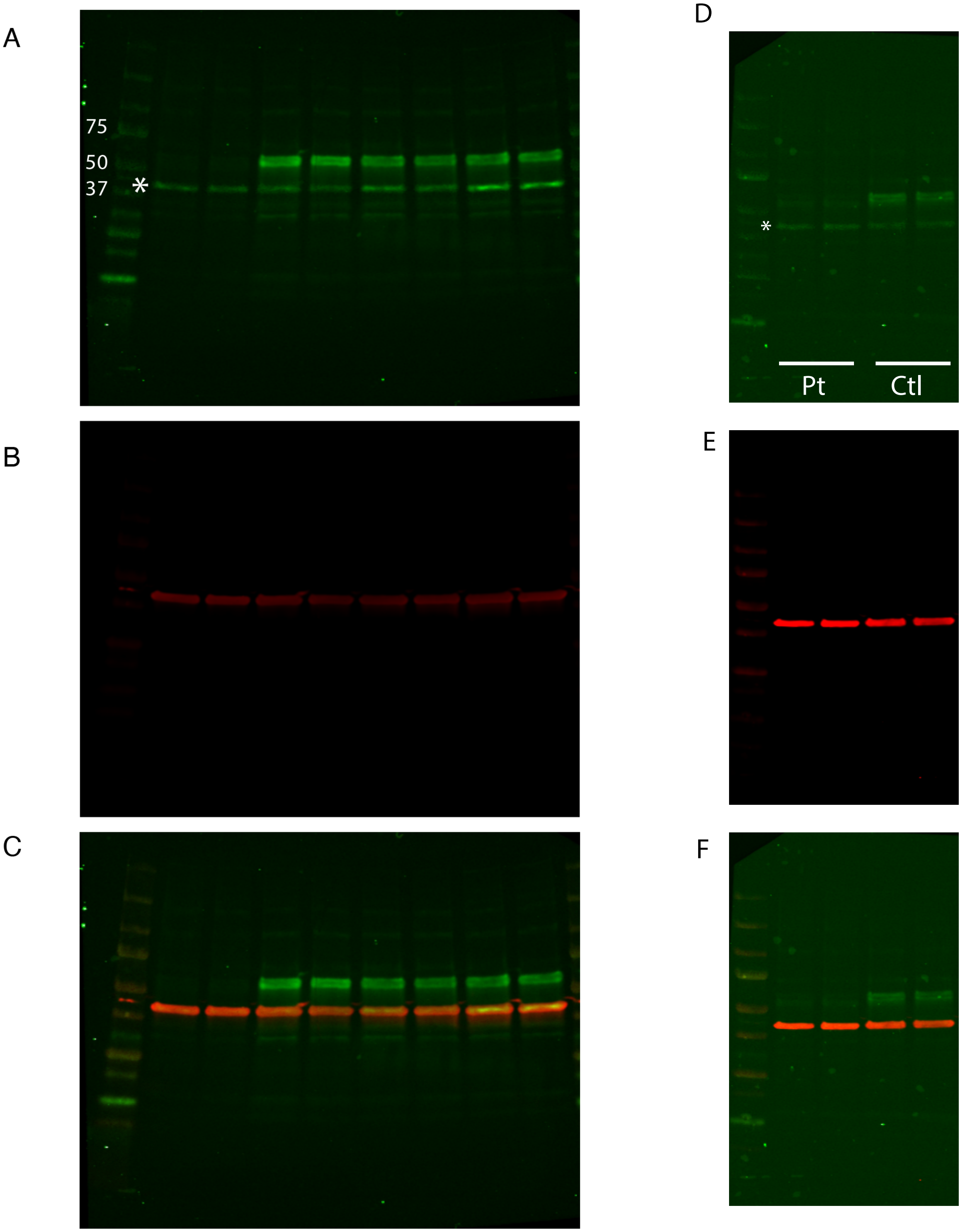

A
75
50
37
*
B
C
D
*
Pt
Ctl
E
F

Supplementary Figure 1. Original, full-length version of the Western Blots with RARS2 antibody.

A-C. Full-length version of the Western Blot presented in Figure 3C. **D-F**. Independent replication of the experiment in Figure 3C with cell lysates from patient (Pt) and control (Ctl) showing a 78% reduction in RARS2 levels in patient compared to control. **A, D.** RARS2 detection was performed with anti-RARS2 (ab230274, Abcam) and then IRDye 800CW Goat anti-Rabbit IgG Secondary Antibody (green). Molecular weights are indicated on the left (kDa). A non-specific band is detected (*) with a molecular weight close to β Actin (42kDa) and lower than RARS2 molecular weight (65 kDa). **B, E.** β Actin was detected with Anti-β Actin (AM4302, Invitrogen) and then IRDye 680RD Goat anti-Mouse IgG Secondary Antibody (red). **C, F.** Merged images of the blots imaged under different acquisition channels.
